# Machine Learning-Based Prediction and Analysis of Chinese Youth Marriage Decision

**DOI:** 10.3390/bs15121750

**Published:** 2025-12-18

**Authors:** Jinshuo Zhang, Chang Lu, Xiaofang Wang, Dongyang Guo, Chao Bi, Xingda Ju

**Affiliations:** 1 School of Psychology, Northeast Normal University, No. 5268, Renmin Street, Nan Guan District, Changchun 130024, China; 2 Jilin Provincial Key Laboratory of Cognitive Neuroscience and Brain Development, Changchun 130024, China; 3 Office of the Party Committee and Administration, Jilin Jianzhu University, No.5088 Xin Cheng Street, Nan Guan District, Changchun 130000, China; 4 Department of Public Security, Jilin Police College, 1399 Boshuo Rd, Jingyue District, Changchun 130000, China

**Keywords:** marriage decision, machine learning, SHAP values, influencing factors

## Abstract

This study investigates the key factors that influence marriage decision among Chinese youth using machine learning techniques. Using data from the China Family Panel Studies (2018–2020), we extracted 1700 samples and filtered 26 significant variables. Seven machine learning algorithms were evaluated, with CatBoost emerging as the most effective. SHAP (SHapley Additive exPlanations) analysis revealed that work-related variables were the most strongly associated with predictions, accounting for 30% of the predictive power, followed by other factors such as demographic and education. Notably, we found that commute time and working hours exceeding 50 min/hours were negatively associated with marriage likelihood, while job satisfactions showed a non-linear relationship with marriage decision. The findings highlight the determinant of work–life balance in marriage decision and the complexity and nonlinear relationship in social decision-making. The objective of this study is to provide scientific data support for policy makers in an era of declining marriage rates in China. This study not only reveals the key factors affecting marriage decision but also provides critical evidence-based support for policymakers to prioritize resource allocation and formulate targeted policies amid declining marriage rates in China.

## 1. Introduction

Since antiquity, marriage has served as a pivotal social contract, offering robust support for individual growth and social cohesion. As an institution, marriage plays a central role in most societies, influencing decisions regarding labor supply, consumption, reproduction, and other significant aspects, thereby receiving considerable attention in academic circles (Grossbard-schectman & Grossbard-Shechtman, 2019). The marriage rate, defined as the ratio of marriages to the total population over a specified period (Gao et al., 2022), is fundamental for maintaining the stable functioning of the marriage institution.

In recent years, the persistent decline in marriage rates in China has garnered substantial societal attention. According to the “China Marriage and Family Report 2023” published by the China Association of Social Security, the number of first marriages in China decreased by 50% from 2013 to 2021, a significant decline within just eight years. Given the adverse effects of the declining marriage rates on social development and their specific manifestations in Chinese society, it is crucial to identify the key factors influencing individuals’ marriage decision, elucidate the underlying causes of the observed decline, and formulate effective population policies accordingly.

Previous research has identified numerous factors influencing marriage decision. Among these, demographic characteristics, including sex, age, and height, play significant roles. Gender differences significantly influence individuals’ entry into marriage (Allendorf et al., 2017; Desai & Andrist, 2010), and individuals of different ages exhibit significant variations in their marriage decision (Mahay & Lewin, 2007). Additionally, studies have demonstrated that individuals with higher educational levels tend to postpone their marriage decision (Lai et al., 2023; X. Li & Cheng, 2019; Liang & Yu, 2022). Furthermore, a notable positive correlation exists between parental educational attainment and the likelihood of their children entering a first marriage (Lan & Kuang, 2022). Wealth and income have also been found to be crucial in marriage decision (Fieder & Huber, 2023; Watson & McLanahan, 2011). Government tax policies also impact individuals’ propensity to marry (Fink, 2020; Michelmore & Lopoo, 2021). Besides these, social culture is another significant determinant of marriage. In China, traditional concepts such as “Marriage of matching doors” and associated social expectations have significantly shaped the Chinese people’s unique perception of marriage (Hu, 2016; Zhou, 2019). 

These studies offer a multidimensional perspective, facilitating a deeper understanding of the intricacies of marriage decision and providing robust theoretical support for a comprehensive understanding of marriage and the formulation of suitable population policies. However, despite existing literature has extensively identified multiple factors influencing marriage decisions, but given the inherent limitation of government resources, prior studies have failed to systematically integrate these multifaceted variables into a unified analytical framework for comparative evaluation. As a result, there is a lack of empirical evidence quantifying the relative importance of different influencing factors and their specific impact patterns, which directly hinders policymakers from identifying priority areas for intervention. Addressing this gap requires a comprehensive approach that synthesizes diverse variables, quantifies their predictive contributions, and clarifies their action mechanisms—an effort essential to providing evidence-based guidance for optimizing the allocation of government resources and formulating targeted population policies. 

Over recent decades, machine learning (ML) techniques and methodologies have witnessed rapid development (Ostroumova et al., 2017). ML models offer a critical advantage over traditional linear models like logistic regression: they inherently capture nonlinear effects and complex interaction effects without requiring manual specification of relationships. In contrast, linear models oversimplify these dynamics by assuming proportional, constant effects, failing to account for the nuanced, context-dependent patterns that define social phenomena (Kyriazos & Poga, 2024). Simultaneously, the accumulation of data has enabled machine learning modeling based on large datasets to demonstrate its effectiveness in addressing diverse fields, including those in healthcare, cybersecurity, and biological sciences (Ghosh & Dasgupta, 2022; Latif et al., 2023; Talaei Khoei & Kaabouch, 2023). Against this backdrop, social science research has also increasingly shifted from traditional deductive methods to more iterative and interactive inductive approaches—unlocking new potential for in-depth exploration in the field (Grimmer et al., 2021). For instance, Lin (2025) employed machine learning to predict consumer behavior, while Hu et al. (2025) used ML to predict friends’ behavior in real-world networks. With regard to the prediction of marital decisions, Moulaei et al. (2024) utilized machine learning algorithms to predict women’s fertility intentions, and Sharma et al. (2021) and Moumen et al. (2024) applied ML to forecast divorce. Employing machine learning algorithms to analyze marriage decision can effectively integrate numerous socioeconomic indicators, handle nonlinear relationships, uncover intricate patterns within datasets, and offer comprehensive and accurate insights into the key factors shaping marriage decision. 

Although previous research has made significant contributions in the field of marriage by using ML techniques, there remain many issues worthy of attention. Existing research has predominantly focused on specific demographic groups, such as women, or relied on conventional statistical techniques, like the linear regression model. More critically, these studies lack substantive explanatory contributions: while some estimate feature importance to identify relevant factors, they fail to leverage interpretive frameworks like SHAP (SHapley Additive exPlanations) (Lundberg & Lee, 2017)—a unified approach to quantifying the relative importance of multi-dimensional determinants and “opening the black box” of ML models for in-depth interpretation. Without such tools, the mechanisms through which socioeconomic and demographic factors interact to influence marriage decisions remain under-explored, and there remains a scarcity of large-scale sample-based predictions and ML-driven data mining for marriage decision research in China.

This study extracted 1700 samples and filtered 26 significant variables from CFPS (China Family Panel Studies) 2018–2020 (Chunni, 2014), the CFPS is publicly accessible on its official platform managed by the Institute of Social Science Survey (2025). Seven machine learning algorithms were evaluated, and a predictive model for marriage decision was developed within the CatBoost framework. Furthermore, SHAP was employed to rank the importance of each variable, and a global interpretation of the model was conducted. The objective of this study is to conduct an objective and comprehensive analysis of the key factors influencing individuals’ marriage decision, identify individual differences among different marriage decision, and provide scientific data support for policymakers. Through a comprehensive analysis of marriage decision, this research not only offers a solid practical foundation for addressing current demographic challenges in society but also serves as a clear research idea for implementing big-data-based research in the social sciences with the aid of machine learning.

## 2. Methodology

### 2.1. Sample Extraction 

**Figure 1:**
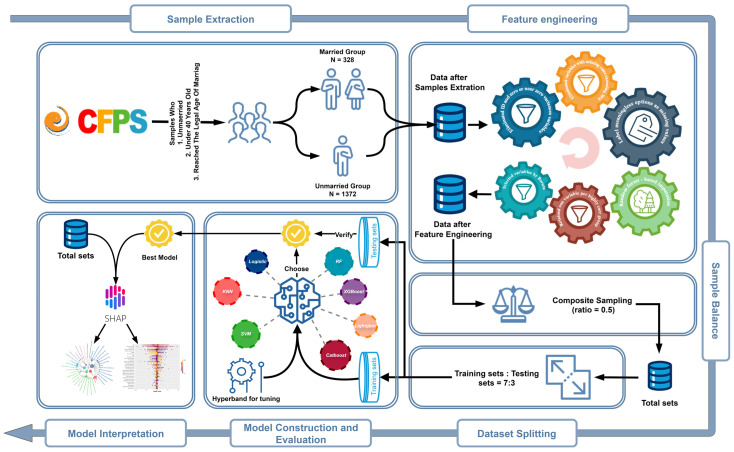
Flowchart.

Figure 1 shows the flow chart of this study. In this study, we used information on unmarried individuals in 2018 as features to predict their marriage status in 2020. In each survey of CFPS, there is a question asking about marital status: “What is your current marital status?” (1. Unmarried, 2. Married, 3. Cohabiting, 4. Divorced, and 5. Widowed). The label was defined as follows: Individuals who were unmarried (status “1”) or cohabiting (status “3”) in 2018 and subsequently transitioned to married (status “2”), divorced (status “4”) or widowed (status “5”) in 2020 were categorized as the married group. In contrast, individuals who were unmarried or cohabiting in both 2018 and 2020 were categorized as the unmarried group. The study sample selection criteria were based on the legal marriage age in China, which is 22 for males and 20 for females. We concentrated on unmarried individuals under 40 in 2018, specifically those born between 1978 and 1996 for males and between 1978 and 1998 for females. 

### 2.2. Feature Engineering 

In light of the intricate nature and robust skip logic of CFPS survey items, which display attributes such as variable redundancy and high levels of missing values. The feature engineering process conducted in this study is as follows:

- 24 ID variables and 843 variables with zero or near-zero variance were eliminated.
- Label options without meaningful research implications as missing values. For example, the label “−8” is used to signify “not applicable”.
- 273 variables with missing rates greater than 30% were eliminated.
- Random forest imputation was used to fill in the missing data (Stekhoven & Bühlmann, 2012). To address potential data leakage risks associated with imputation timing, we compared model performance between two protocols: imputation prior to dataset splitting (the approach adopted in this study) and imputation after splitting. Detailed results of this comparative analysis are presented in Appendix A Table A1, which shows minimal differences across key metrics (AUC, precision, recall, F1-score) between the two protocols—confirming that the impact of potential leakage is negligible.
- Among the groups of highly correlated variables (Pearson’s correlation coefficient & Cramér’s V coefficient greater than 0.75), only one variable was retained in each group. 36 variables were eliminated.
- The Boruta algorithm was used for feature selection, 26 variables were eliminated. (Kursa et al., 2010).

### 2.3. Sample Balance 

The dataset processed after the above steps has a sample imbalance problem, with a ratio of married and unmarried samples of 1:4 (328:1372). In order to reduce the impact of the sample imbalance on the prediction model, we used the R package “ROSE” (Lunardon et al., 2014) to implement composite sampling for sample balancing (composite ratio = 1:1). Resulting in a post-balancing married-to-unmarried ratio of 1:1, which will ensure the reliable model performance. To verify the effectiveness of sample balancing, we compared the performance of all seven machine learning models on both the original unbalanced and balanced training datasets. Detailed comparative results are presented in Appendix A Table A2.

### 2.4. Dataset Splitting 

Previous research had demonstrated that in the field of machine learning, it is essential to split the dataset into training and testing sets when discovering patterns and building predictive models (Joseph & Vakayil, 2022; H. Liu et al., 2019). This approach not only validates the model’s generalization ability, but also prevents overconfidence in the results due to data leakage. In this study, we randomly selected 70% of the samples from the married and unmarried groups, respectively, as a training set and the rest of the samples as an independent test set. 

### 2.5. Model Construction and Evaluation 

In this study, seven machine learning algorithms were employed: logistic regression, k-nearest neighbors (KNN) (Cover & Hart, 1967), support vector machine (SVM) (Cortes & Vapnik, 1995), random forest (RF) (Breiman, 2001), extreme gradient boosting (XGBoost) (T. Chen & Guestrin, 2016), light gradient boosting machine (LightGBM) (Ke et al., 2017), and categorical boosting (CatBoost) (Ostroumova et al., 2017). In order to refine the models and enhance their predictive accuracy, we employed the Hyperband algorithm (L. Li et al., 2018) for parameter tuning (Appendix A Table A3).

The performance of the model was evaluated on the training set using a 10-fold cross-validation during the evaluation comparison phase after model construction. 10-fold cross-validation is a widely used and validated model evaluation method in machine learning. Its primary objective is to improve the generalizability of the model and reduce randomness and bias in the evaluation results, thus providing more robust performance estimates (Refaeilzadeh et al., 2009). To comprehensively assess model performance, we used a variety of evaluation metrics, including AUC (area under the ROC curve), accuracy, precision, recall, specificity, and F1 score. These metrics will provide us with an intuitive basis for model comparison. We used AUC as the main evaluation metric during model training.

The processes of model construction, parameter tuning, and evaluation in this study were carried out within the MLR3 package family of the R language (version 4.3.2) (Lang et al., 2019; R Core Team, 2023).

## 3. Results

### 3.1. The Association of Marriage Decision with Variables

**Figure 2:**
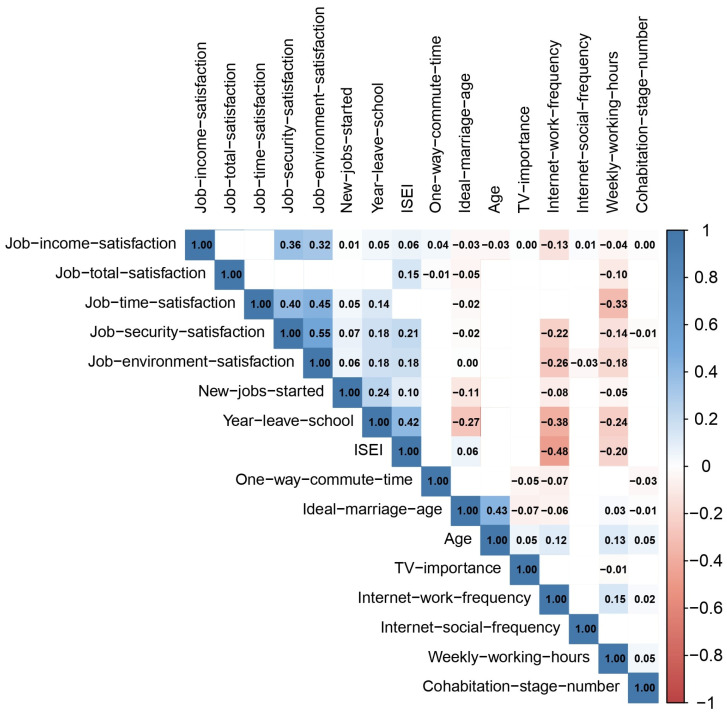
Correlation Plots for Continuous Variables.

A preliminary statistical analysis was performed on the dataset. There were 895 men and 805 women, resulting in a male-to-female ratio of 52.6% to 47.4%. The mean age was 25.48 years (SD = 4.18). To further explore the differences between the married and unmarried populations, independent samples *t*-test were carried out on 16 continuous variables (refer to Table 1). The results showed significant differences between the two groups in seven variables: age, number of cohabitation stages, ideal age for marriage, frequency of Internet use for socializing, frequency of Internet use for work, number of new jobs, and the importance of TV. The effect sizes (Cohen’s d) for the number of cohabitation stages were the most significant, exceeding 0.29, while the effect sizes for the remaining variables ranged from 0.16 to 0.2. Specifically, the married group was older, had experienced more cohabitation stages, had a younger ideal age for marriage, used the Internet more frequently for work but less frequently for socializing, had fewer new jobs, and regarded TV as a more important source of information. Additionally, Pearson’s correlation coefficients were computed for these continuous variables, and correlation diagrams were plotted to evaluate the relationships and their directions (refer to Figure 2). 

For categorical variables, chi-squared test were employed to compare the differences between the married and unmarried groups (refer to Table 2). The results indicated significant differences for all variables except gender. Specifically, the main type of job was significant at the 0.05 level; the variables of whether computers were used at work and whether endowment insurance was held were significant at the 0.01 level; and the remaining six variables—schooling status, whether other education experience was present, whether in a relationship, the highest level of education completed, whether full-time work experience was available, and whether financial help from fathers was received—were all significant at the 0.001 level.

### 3.2. Comparison of the Machine Learning Model Performance 

**Figure 3:**
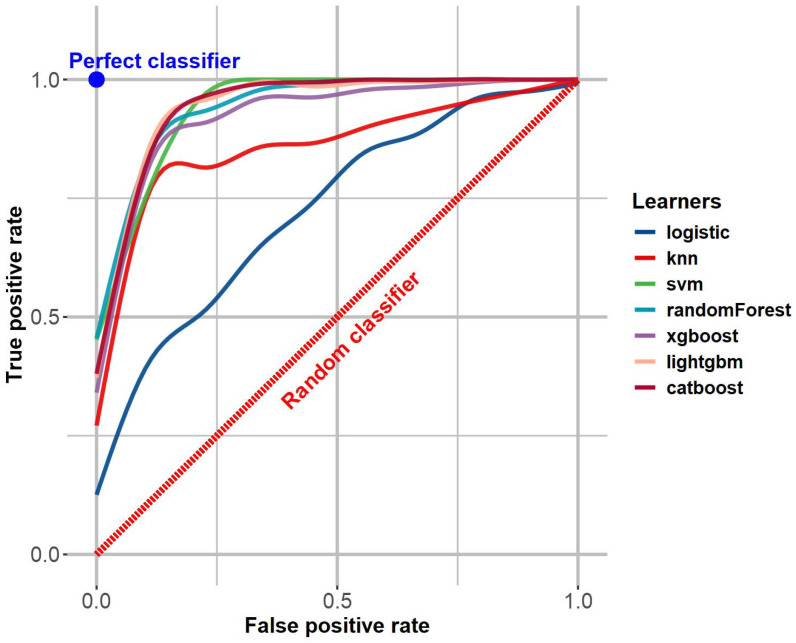
ROC Curves for Machine Learning Models.

The performance of seven machine learning models was rigorously evaluated using multiple metrics (refer to Table 3). The logistic model performed poorly, with its accuracy never exceeding 0.7 and an AUC of 0.73/0.76, which fell short of the generally accepted practical threshold of 0.8 (Çorbacıoğlu & Aksel, 2023). In contrast, the performance of the KNN model was within the normal range, with both the AUC value and F1 score ranging from 0.8 to 0.9. The SVM and the remaining four integrated algorithms (RF, XGBoost, LightGBM, CatBoost) demonstrated the best performance, with AUC values exceeding 0.93 and F1 scores exceeding 0.85 in the training set. Figure 3 shows the ROC curves for the models. The performance of the CatBoost model was further evaluated in the test set, yielding an AUC value of 0.95. This validates the claim that this model exhibits robust generalization ability and is not prone to significant overfitting.

### 3.3. SHAP-Based Importance Ranking of Variables & Aspects of Marriage Decision 

**Figure 4:**
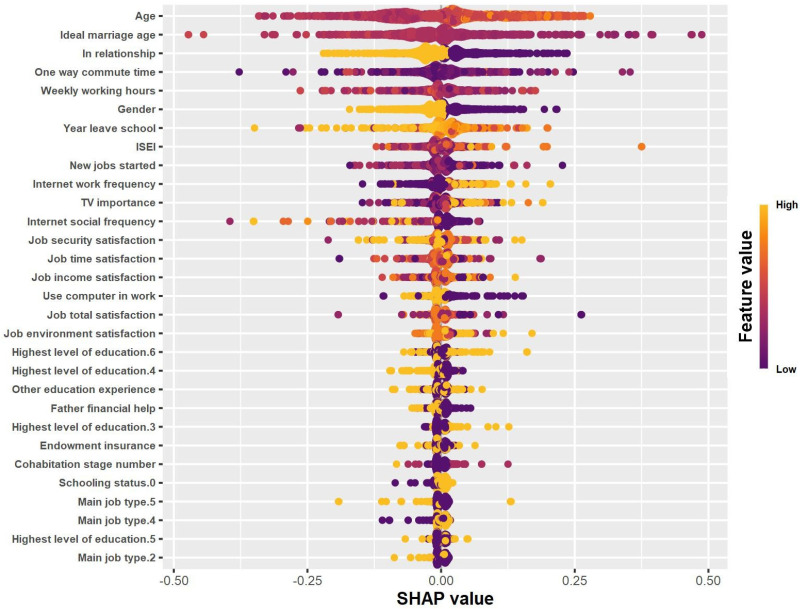
Summary Plot of the Bee swarm.

During the model interpretation phase of this study, the SHAP values were computed using the established CatBoost model. SHAP (SHapley Additive exPlanations) is a method for interpreting machine learning model predictions, based on Shapley values in cooperative game theory. It aims to quantify the contribution of each feature to the model’s prediction results by assigning a numerical value to each feature of each sample, which reflects the feature’s importance and impact in the prediction process. To clarify the sign convention adopted in this study: Positive SHAP values push the prediction towards ’Married’, while negative SHAP values push it towards ‘Unmarried’. This convention is consistently applied in all subsequent SHAP-based analyses. Furthermore, in this paper, the acronym MSV is introduced in place of mean|SHAP value| for clarity and consistency, and this acronym will be utilized in the subsequent sections of the paper to denote this specific metric. The size of MSV directly reflects the overall importance of a feature in the marriage decision prediction model: A higher MSV indicates that, on average, the feature exerts a stronger association with the model’s prediction results across all observed samples—meaning the feature plays a more critical role in distinguishing between “Married” and “Unmarried” outcomes. Conversely, a lower MSV suggests the feature has a relatively weaker average impact on predictions. Figure 4 displays the summary plot of the bee swarm, where each point for each feature represents a sample. The colors represent the raw values of their features, and the position of each point on the x-axis indicates the magnitude of its SHAP value.

**Figure 5:**
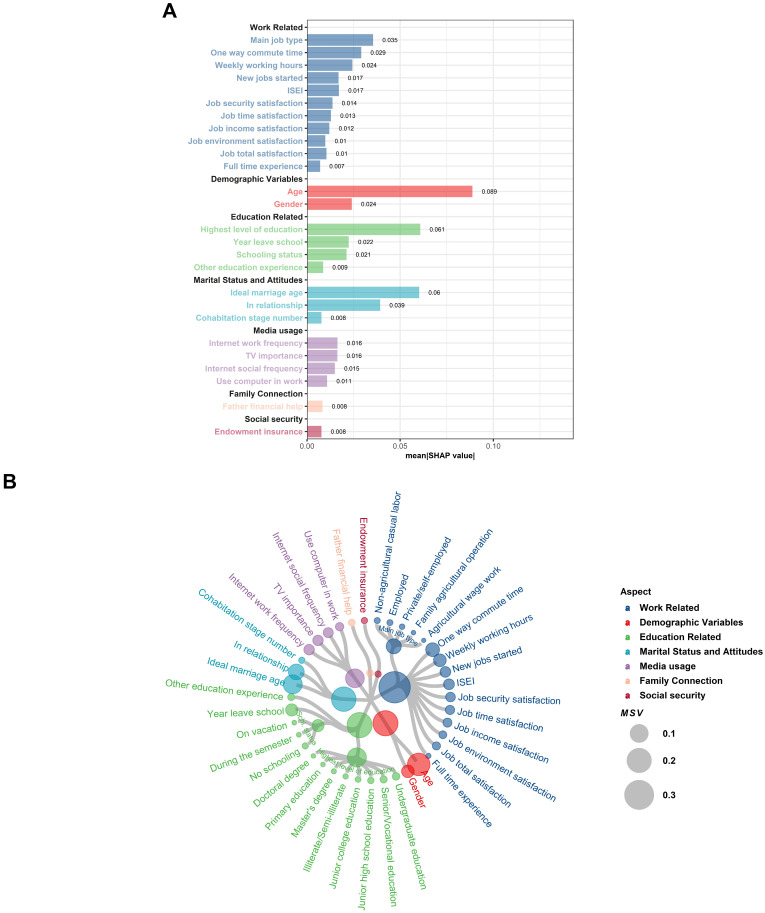
Importance Ranking of Variables after Categorization and Aggregation. Subplot (**A**) presents a bar chart of the mean SHAP values (MSV) per variable, reflecting their average contribution to marriage decision prediction. Different colors denote distinct variable categories (e.g., blue for work-related factors and red for demographic factors). Subplot (**B**) is a circular dendrogram that visualizes the hierarchical relationships and contributions of predictive factors, with circle size proportional to the respective variable’s MSV. The color coding aligns with that used in Subplot (**A**).

Figure 5A illustrates the MSV of each variable, represented after categorizing the variables and ranking them according to their impact on the results. The total MSV for the 26 variables was 0.596. Figure 5B is a circular dendrogram plot, which is used to visualize multiple variables for predicting marriage. In the plot, different colors denote different categories of variables. For instance, blue represents work-related variables, and red represents demographic variables. The size of each point reflects the magnitude of the SHAP value; the larger the point, the stronger the association. Regarding individual variables, age was identified as the most critical predictor of marriage decision, with a significant MSV of 0.089. The highest level of education completed, the ideal age of marriage, relationship status, and the main type of job ranked second to fifth, with an MSV range of 0.039−0.061. This indicates that the MSV of these five most prominent variables accounts for more than 47.8% of the total MSV. The variables ranked sixth to tenth were commute time, weekly work hours, gender, year of leaving school, and schooling status. These variables also showed an MSV of more than 0.02. Due to space limitations, the remaining 16 variables will not be fully presented here. 

A comparative analysis across different aspects revealed that, although no variables had an extremely high MSV like age, the 11 work-related variables were the most significant aspect, with an MSV of 0.189. Collectively, these variables accounted for approximately 30% of the total MSV. Among these variables, five were related to job satisfaction, while the others were related to aspects such as ISEI (International Socioeconomic Index) and work type. The next aspect to consider was the demographic variables, including age and gender, which are two classical variables of interest in the social sciences. Although the number of variables in this aspect was relatively small, their individual MSV could reach the first and eighth positions among all variables, respectively, with a total MSV of 0.113. Following that were four education-related variables: the highest level of education completed, year of leaving school, schooling status, and whether other education experience was present. The combined MSV for these variables was 0.113. In fourth place was marital status and attitudes, which included three variables: the ideal age of marriage, relationship status, and the number of cohabitation instances. The total MSV was 0.107. Four media-related variables were also of interest: computer use at work, Internet use for socializing and work, and the importance of television, with a total MSV of 0.058. The last two aspects, family connection and social security, each had only one variable, namely whether financial help from the father was received and whether endowment insurance was held, with MSV values of 0.08, respectively. 

### 3.4. Nonlinear Variable Dependency Relations and Association Patterns 

**Figure A1:**
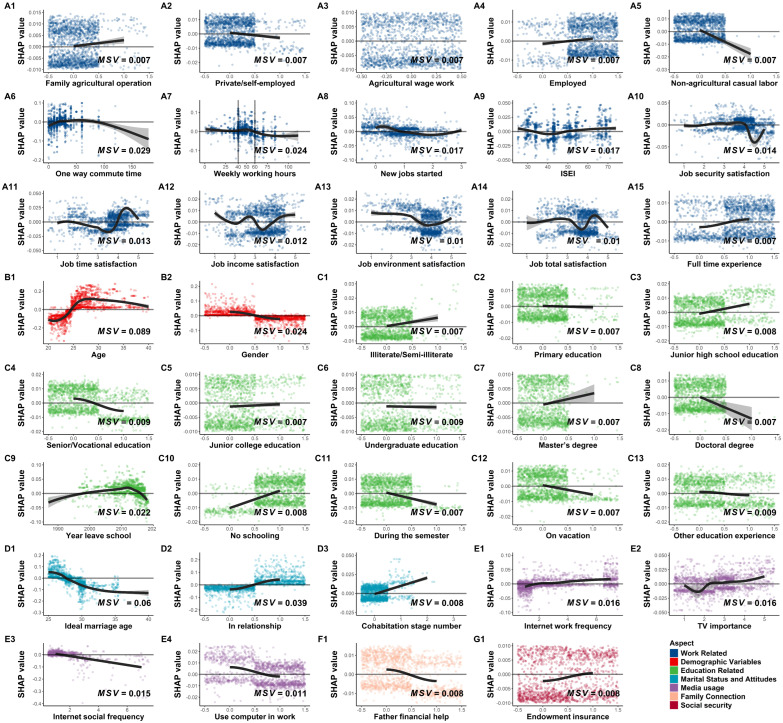
Variable dependence graphs for all variables. Different colors represent various categories: blue indicates work related categories, including various aspects of employment and job satisfaction metrics. Red represents demographic variables, specifically age and gender. Green signifies educational categories, covering the highest level of education attained and schooling status. Turquoise illustrates marital status and attitudes, such as ideal marriage age and current relationship status. Purple highlights variables related to internet usage, while orange represents financial assistance variable (whether one receives financial help from their father), and red indicates social security variable (whether one has endowment insurance). Note: (a) Fitting method differences: For subplots (**A1**,**A2**,**A4**,**A5**,**C1**–**C3**,**C5**–**C8**,**C10**–**C12**,**D3**,**E3**), Loess fitting failed, so linear regression was used instead. Subplot A3 had too few valid samples (only two), and after sample balancing, no valid samples remained. (b) Dichotomous variables: In subplots (**A1**–**A5**,**A15**,**B2**,**C1**–**C8**,**C10**–**C13**,**D2**,**E4**,**F1**,**G1**), the variables are dichotomous. In subplot (**B2**), 1 represents male and 0 represents female; elsewhere, 1 indicates “yes” and 0 indicates “no”.

To understand how individual features are associated with the model’s predictions, We have drawn the variable dependency graph. Each point represents a sample, where the horizontal coordinate indicates the level of that sample in this feature, and the vertical coordinate indicates the corresponding SHAP value—a straightforward metric: positive values mean the feature pushes the sample toward a “married” prediction, while negative values push it toward “unmarried”. The black line in the figure is the curve fitted to the scatterplot using the Locally Weighted Linear Regression (LOESS) method, and it also shows the confidence interval at the 0.95 level. To enhance the plot’s readability, a random perturbation ranging from −0.5 to 0.5 was added to each point in the horizontal direction. The variable dependence for all variables was plotted in Appendix A (Figure A1).

**Figure 6:**
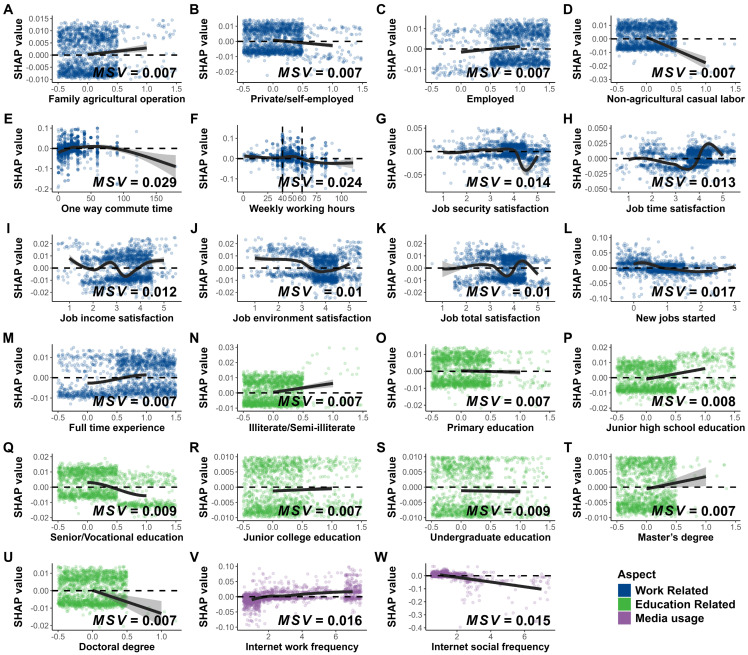
Variable Dependence Graphs. Different colors indicate various categories: blue for work related variables, including employment types, job satisfaction metrics, and work conditions and changes; green for educational variables, covering different education levels; and purple for media usage, including two variables for internet usage in different environments. Note: (a) Fitting method differences: For subplots (**A**–**D**,**N**–**P**,**R**–**U**), Loess fitting failed, so linear regression was used instead. (b) Dichotomous variables: In subplots (**A**–**D**,**M**–**U**), the variables are dichotomous, 1 indicates “yes” and 0 indicates “no.”.

Figure 6A–D present a series of variable dependency plots for the main job type. As depicted in the figures, the SHAP values of the samples engaged in family agricultural operations and those who were employed were higher than those of other two work types. In contrast, the average SHAP scores of the samples categorized as private/self-employed and non-agricultural casual labor were lower. E and F illustrate the variable dependencies of one-way commute time and hours worked per week on the corresponding SHAP values, respectively. As evident from the figures, the relationships between these two variables and the SHAP values exhibit a stable-decline curve, with the stabilization of the SHAP values persisting up to approximately 50. Specifically, within the range where the commuting time is less than 50 min and the weekly working hours are less than 50, the average SHAP value shows a subtle change as the independent variable increases. Subsequently, as the level of the independent variable continues to rise, the average SHAP value decreases. G-K show the dependency graphs of five job satisfaction-related variables. The predictive impacts of these variables on marriage do not show a simple linear relationship. Instead, they display a localized “v”-shaped curve at medium to high levels of the independent variable. L and M present variable dependency plots for two job-stability-related variables. The plots reveal a clear downward trend in SHAP values as the number of newly started jobs increases, and samples with full-time work experience exhibit higher SHAP values. N-U display the variable dependence plots for the highest educational attainment. The plots indicate that samples with the lowest educational levels (illiterate/semi-literate and junior high school education) have the highest SHAP values, whereas samples with higher educational levels (senior/vocational education and doctoral degree) have significantly lower SHAP values compared to the average. V and W present the variable dependence graphs of Internet use frequency, which are divided into two sections: Internet use frequency for work and for socializing. The graphs illustrate that the trends of these two sections are diametrically opposed. As the Internet use frequency increases, the SHAP value in the work-related scenario gradually increases from negative to positive, while the SHAP value in the social-related scenario gradually decreases from positive to negative.

## 4. Discussions

In the results section, seven machine learning models were evaluated, and CatBoost was found to perform optimally. Through SHAP analysis, the importance of various variables in predicting marriage decision was identified. Based on these findings, upon categorizing the variables, it was found that work-related variables dominated marriage prediction, accounting for approximately 30% of the total MSV. Work serves not only as an economic source but also is associated with an individual’s social status, self-identity, and social circle. Demographic variables had the second-strongest association with marriage decision, illustrating the association of individual physiological and psychological maturity on such decisions. The education-related variables had a combined MSV comparable to that of demographic variables, reflecting the indirect effect of education on marriage decision via its association with individual values, career choices, and other aspects. Marital status and attitude-related variables indicated that individuals’ expectations regarding marriage, relationships status, and cohabitation experiences were associated with the likelihood of marriage. Although the MSV of media use, family connection, and social security-related variables was low, they still demonstrated a potential impact on individuals’ marriage decision, highlighting the role of the modern media environment, family support, and social security in the context of marriage decision. 

This study identified work as the most significant factor associated with individuals’ marriage decision. This finding is consistent with previous research (Dew & Price, 2011). Results regarding the main job type indicated that samples engaged in family agricultural operations and those who were employed, due to their greater stability and economic security, had a higher likelihood of marriage compared to samples of private/self-employed individuals and non-agricultural casual laborers. Similarly, results for variables related to job stability showed that samples with stable full-time jobs were more likely to be married. This result aligns with previous research findings (Ahituv & Lerman, 2011); specifically, the more stable the job, the higher the likelihood of marriage. 

Regarding job satisfaction, it presents complex non-linear results. These results indicate that, contrary to initial expectations, the relationship between job satisfaction and the likelihood of marriage is not monotonically increasing. Specifically, a localized “v”-shaped curve is observed at medium to high levels of the independent variable. One plausible explanation is that when job satisfaction is at a moderate level, individuals’ physiological and security needs are partially satisfied. However, recent developments in China’s employment landscape have been less than promising (Tang & Shi, 2017), and upward mobility has become a significant challenge for young professionals (Schucher, 2017). In this highly competitive environment, individuals are motivated to prioritize career advancement, which can be associated with a suppression of their socialization needs. As a result, individuals at this stage are the least likely to marry. Conversely, when job satisfaction reaches a very high level, individuals may have achieved substantial career progress, accompanied by a reduction in stress. At this point, they may start considering marriage and family life, seeking fulfillment and balance in their emotional and personal spheres. Thus, an increase in job satisfaction can also be associated with a corresponding increase in the likelihood of marriage. 

Time-related results revealed that the likelihood of marriage decreased rapidly when the one-way commute time or weekly working hours exceeded 50 units. This phenomenon can be effectively explained by the work-family conflict theory (Clark, 2000). Once the commute time or working hours surpassed the threshold of 50, work-family conflict intensified, resulting in increased stress-related conflicts. These heightened conflicts could ultimately decrease the likelihood of marriage, as individuals might struggle to allocate sufficient time and energy to foster intimate relationships while managing their work responsibilities (Y. Chen et al., 2020; Golden & Wiens-Tuers, 2006; Tomono et al., 2021). And it also underpins the interpretation of work-related variables (accounting for 30% of total MSV). These findings on work-related variables aligning with the theory’s core proposition that “work overload disrupts non-work domain functioning”—when youth spend excessive time on commuting or work, they lack the capacity to foster intimate relationships, thus reducing marriage propensity. Moreover, research has shown that variables other than work-related ones can also significantly associated with individuals’ marriage decision. Marital status and attitude variables, such as the ideal age of marriage, demonstrate the substantial impact of cultural and social values on marital choices. The significance of these variables suggests that marriage is not only determined by individual preferences but also reflects social and cultural expectations (Aniciete & Soloski, 2011; Estin, 2011). Educational variables have also been shown to have a considerable association with marriage decision. However, our results deviate from previous studies (B. Liu & Liu, 2018). The variable dependency graph indicates that the decision to pursue higher education does not affect an individual’s marriage decision. Instead, attaining only compulsory education seems to increase the likelihood of marriage, while completing upper secondary education appears to decrease it. A possible explanation for this is that the majority of samples in this study belong to the generation that benefited from China’s tertiary expansion policy. For this generation, the economic and social advantages conferred by higher education are no longer as prominent (Zhang et al., 2023). Additionally, it is important to note that the impact of Internet use on marriage varies across different contexts. Our hypothesis posits that frequent Internet use at work may facilitate the expansion of professional networks and quick access to information, thereby enhancing an individual’s socioeconomic status and subsequently increasing the likelihood of marriage. Conversely, excessive reliance on the Internet in social contexts may restrict face-to-face interpersonal interactions and diminish opportunities for building deep relationships, thus reducing the probability of marriage. These disparities highlight the intricate association of Internet use on marriage decision within diverse social environments.

However, there are also several findings that deviate from previous studies. Variables related to income and economic situation do not exhibit a remarkable level of importance in terms of both the number of variables and the total MSV (Kuo & Raley, 2016; Yu & Kuo, 2017). On one hand, economic status may not directly and solely determine the decision to marry but is instead indirectly reflected through work-related variables. The nature of an individual’s job and career progression typically determine their level of economic income (Janietz, 2024), meaning that job-related variables already capture some of the effects of economic factors on marriage decision to a certain extent. On the other hand, this can be attributed to the association of Chinese traditional culture, which emphasizes stability and a reasonable standard of living (Ge et al., 2023). In this cultural context, the significance of the workplace, job type, and job satisfaction is amplified.

Culture-adapted extension of Marriage Market Theory (adapted from Becker, 1973, 1974) contextualizes associations involving education, family background, and traditional attitudes. In China’s marriage market, where “marriage of matching doors” (Hu, 2016) and stability-oriented values predominate, variables including parental financial support, educational attainment, and ideal marriage age reflect cultural norms defining “contextually appropriate” marital candidates. The nonlinear association between education and marriage likelihood observed in this study should be interpreted as a cohort-specific and context-bound pattern rather than a general judgment on educational value: the majority of our samples belong to the generation that benefited from China’s tertiary education expansion (Zhang et al., 2023), where the once-prominent “status premium” of higher education in the marriage market has become more stratified. For this cohort, youth with compulsory education (e.g., junior high school) may align with certain regional or group-specific traditional norms of “early life stability”, leading to a relatively higher observed marriage likelihood in our sample; in contrast, youth with senior/vocational education often face a “transitional phase” of prioritizing career advancement to meet cultural expectations of “economic stability” before marriage—this delay is a reflection of structural career pressures rather than a negative outcome of education itself. Notably, this pattern does not negate the long-term value of higher education, nor does it suggest that compulsory education is “advantageous” for marriage in a universal sense; instead, it reflects the complex interaction between educational attainment, cohort characteristics, and cultural marital norms in contemporary China.

Based on all the research findings, we had integrated Work-Family Conflict Theory and culture-adapted Marriage Market Theory, we find that Chinese youth’s marriage decisions are shaped by a “balance between structural work demands and cultural marital norms”: work variables (guided by Work–Life Balance Theory) determine the “resource capacity” (e.g., time, emotional energy) for investing in marriage, while education, family, and attitude variables (informed by the culture-adapted Marriage Market Theory) determine the “cultural congruence” of individuals with context-specific marital expectations. This dual-process explanation addresses the ad hoc nature of individual findings, highlighting that marriage decisions are not random associations but coherent outcomes of structural constraints (e.g., work overload) and cultural contexts (e.g., norms of stability).

### 4.1. Practical Implications

Drawing on the key findings and theoretical frameworks, as well as existing policy practices, we propose targeted, actionable policy implications to address declining marriage rates among Chinese youth, with a focus on work. First, urban planning could prioritize “job-housing balance” in new urban districts, such as Beijing Municipal Administrative Center plan that relocates government offices and enterprises to reduce cross-city commutes, which directly responds to the identified 50-min commute threshold. Second, strengthen the enforcement of labor regulations, For example, the “Special Action Plan to Boost Consumption” issued by the Chinese government in 2025 emphasizes that “working hours for employees must not be unlawfully extended.” (Central People’s Government of the People’s Republic of China, 2025). This aligns with the finding that weekly working hours over 50 units are negatively associated with marriage propensity. 

Internet usage is another problem that urge to concern. The potential for a healthier online environment to promote marriage decisions is significant, and improving this environment is highly feasible. The government could launch public campaigns to counter malicious negative marriage discourse. And short video platforms should strengthen their review of videos that maliciously disparage marriage and partners during the content screening process. This aims to reduce the cultural stigma surrounding marriage.

### 4.2. Limitations & Future Research 

It is recognized that this study has limitations that can be addressed in future research. First, only the 2018–2020 sample was modeled. Exploring this data over a longer time period could help identify the developmental patterns of marriage decision among young Chinese individuals. Second, modeling with datasets from multiple countries could lead to more generalizable conclusions. Third, due to space constraints, the non-linear relationships in the dependency graphs of the remaining variables could not be presented and discussed in this article. Future studies should further investigate these relationships. Fourth, as a predictive framework, the study focuses on associative patterns rather than causal estimates, leaving room for future research to clarify causal relationships between key factors and marriage outcomes.

## 5. Conclusions

This study accurately predicts the marriage decision of contemporary Chinese youth by constructing machine learning models based on a large dataset. Using SHAP, the importance of different variables and their patterns of association were quantified. Based on the analysis of the results, it is proposed that future policy-making should prioritize strengthening the job market, specifically by increasing job opportunities and enhancing labor security to boost the likelihood of stable full-time employment. Second, commuting time and working hours can be reduced to less than 50 units by optimizing urban resource allocation, promoting the private economy, and enforcing labor regulations. Additionally, efforts should be made to guide public opinion against excessive involution and the demonization of marriage. This study offers a blueprint for implementing big-data-based research in the social sciences using machine learning. It also provides a clear and actionable framework for future research. 

## Figures and Tables

**Table 1:** Independent Samples *t*-test Result.

Variable	M(SD)	*t*	*df*	*p*	*d*	95% CI
Age	25.48 (4.18)	−2.7	1698	0.007 **	−0.17	[−0.29, −0.05]
Cohabitation stage number	0.08 (0.28)	−4.65	1698	<0.001 ***	−0.29	[−0.41, −0.17]
Ideal marriage age	28.15 (3.24)	3.24	1698	0.001 **	0.2	[0.08, 0.32]
Internet social frequency	1.43 (1.07)	3.12	1698	0.002 **	0.19	[0.07, 0.31]
Internet work frequency	2.79 (2.19)	−2.83	1698	0.005 **	−0.17	[−0.29, −0.05]
ISEI (International Socioeconomic Index)	45.69 (13.81)	0.05	1698	0.96	0	[−0.12, 0.12]
Job environment satisfaction	3.75 (0.85)	1.26	1698	0.208	0.08	[−0.04, 0.20]
Job income satisfaction	3.33 (0.85)	−0.51	1698	0.611	−0.03	[−0.15, 0.09]
Job security satisfaction	3.9 (0.8)	−0.03	1698	0.973	0	[−0.12, 0.12]
Job time satisfaction	3.6 (0.93)	1.27	1698	0.203	0.08	[−0.04, 0.20]
Job total satisfaction	3.63 (0.76)	0.47	1698	0.636	0.03	[−0.09, 0.15]
New jobs started	0.89 (0.74)	2.58	1698	0.010 **	0.16	[0.04, 0.28]
One way commute time	23.07 (19.12)	−0.32	1698	0.748	−0.02	[−0.14, 0.10]
TV importance	2.56 (1.22)	−2.78	1698	0.006 **	−0.17	[−0.29, −0.05]
Weekly working hours	48.88 (15.42)	−0.38	1698	0.706	−0.02	[−0.14, 0.10]
Year leave school	2012.35 (5.1)	1.66	1698	0.097	0.1	[−0.02, 0.22]

Note. ** *p* < 0.01, *** *p* < 0.001.

**Table 2:** Chi-Squared Test Result.

Variable	*χ* ^2^	*df*	*p*
Gender	2.63	1	0.105
Endowment insurance	9.85	1	0.002 **
Use computer in work	8.62	1	0.003 **
Main job type	10.26	4	0.036 *
Schooling status	44.29	2	<0.001 ***
Other education experience	13.31	1	<0.001 ***
In relationship	74.09	1	<0.001 ***
Highest level of education	28.66	7	<0.001 ***
Full time experience	37.98	1	<0.001 ***
Father financial help	25.66	1	<0.001 ***

Note. * *p* < 0.05, ** *p* < 0.01, *** *p* < 0.001.

**Table 3:** Model Performance Metrics.

Stage	Learner	Auc	Acc	Precision	Recall	Specificity	F1
Training	Logistic	0.73	0.65	0.67	0.64	0.67	0.65
	KNN	0.86	0.84	0.89	0.8	0.89	0.84
	SVM	0.94	0.9	0.83	1	0.78	0.91
	RF	0.95	0.87	0.89	0.85	0.89	0.87
	XGBoost	0.93	0.86	0.89	0.83	0.89	0.86
	LightGBM	0.95	0.86	0.88	0.84	0.88	0.86
	CatBoost	0.95	0.86	0.89	0.83	0.89	0.86
Testing	Logistic	0.76	0.67	0.69	0.66	0.69	0.68
	KNN	0.89	0.85	0.89	0.82	0.89	0.85
	SVM	0.94	0.90	0.84	1.00	0.79	0.91
	RF	0.96	0.86	0.86	0.89	0.84	0.87
	XGBoost	0.94	0.86	0.88	0.85	0.87	0.86
	LightGBM	0.95	0.86	0.87	0.86	0.86	0.86
	CatBoost	0.95	0.86	0.87	0.87	0.86	0.87

## Data Availability

The research code will be available in Github at https://github.com/zhangjs-NENU/Public-Code (accessed on 30 August 2025) as open access from the date of publication. CFPS is a restricted database. I am not authorized to publish raw files or process datasets here. For data files, please visit the official website https://cfpsdata.pku.edu.cn/ (accessed on 30 August 2025).

## Appendix A

**Table A1:** Model Performance Comparison on Pre-Split and Post-Split Imputation.

Imputation Type	Learner	Auc	Acc	Precision	Recall	Specificity	F1
Total	Logistic	0.76	0.67	0.69	0.66	0.69	0.68
	KNN	0.89	0.85	0.89	0.82	0.89	0.85
	SVM	0.94	0.90	0.84	1.00	0.79	0.91
	RF	0.96	0.86	0.86	0.89	0.84	0.87
	XGBoost	0.94	0.86	0.88	0.85	0.87	0.86
	LightGBM	0.95	0.86	0.87	0.86	0.86	0.86
	CatBoost	0.95	0.86	0.87	0.87	0.86	0.87
Separate	Logistic	0.75	0.68	0.68	0.72	0.64	0.70
	KNN	0.87	0.79	0.84	0.74	0.85	0.79
	SVM	0.94	0.90	0.84	1.00	0.79	0.91
	RF	0.96	0.87	0.86	0.90	0.84	0.88
	XGBoost	0.90	0.83	0.86	0.81	0.86	0.83
	LightGBM	0.95	0.85	0.87	0.85	0.86	0.86
	CatBoost	0.95	0.86	0.87	0.86	0.86	0.86

**Table A2:** Model Performance Comparison on Unbalanced and Balanced Training Datasets.

Stage	Learner	Auc	Acc	Precision	Recall	Specificity	F1
Unbalance	Logistic	0.59	0.77	0.81	0.93	0.10	0.86
	KNN	0.62	0.81	0.81	1.00	0.00	0.89
	SVM	0.71	0.81	0.81	1.00	0.00	0.89
	RF	0.66	0.81	0.81	1.00	0.00	0.89
	XGBoost	0.72	0.81	0.82	0.99	0.09	0.89
	LightGBM	0.71	0.80	0.82	0.97	0.10	0.89
	CatBoost	0.59	0.77	0.81	0.93	0.10	0.86
Balance	Logistic	0.73	0.65	0.67	0.64	0.67	0.65
	KNN	0.86	0.84	0.89	0.8	0.89	0.84
	SVM	0.94	0.9	0.83	1	0.78	0.91
	RF	0.95	0.87	0.89	0.85	0.89	0.87
	XGBoost	0.93	0.86	0.89	0.83	0.89	0.86
	LightGBM	0.95	0.86	0.88	0.84	0.88	0.86
	CatBoost	0.95	0.86	0.89	0.83	0.89	0.86

**Table A3:** Parameter Ranges and Selected Values.

Learner	Parameter	Value Range	Selected Values	AUC Before	AUC After	Epoch
Logistic	epsilon *	[1 × 10^−12^, 1 × 10^−6^]	2.90 × 10^−12^	0.73	0.73	301
	maxit	[10, 1000]	15.6			
KNN	k	[1, 10]	5	0.83	0.86	35
	distance	[0, 10]	0.03			
SVM	cost	[0.1, 10]	1.25	0.94	0.94	301
	gamma	[0, 5]	0.75			
RF	alpha	[0.1, 1]	0.57	0.95	0.95	14
	max.depth	[1, 30]	28			
	num.threads	[1, 20]	12			
	num.trees	[200, 1500]	375			
XGBoost	alpha *	[1 × 10^−3^, 1 × 10^3^]	1.10 × 10^−3^	0.77	0.93	599
	colsample_bylevel	[0.1, 1]	0.86			
	colsample_bytree	[0.1, 1]	0.49			
	eta *	[1 × 10^−4^, 1]	0.01			
	lambda *	[1 × 10^−3^, 1 × 10^3^]	3.16			
	max_depth	[1, 30]	15			
	min_child_weight	[1, 10]	3.83			
	nrounds	[16, 2048]	2048			
	subsample	[0.1, 1]	0.9			
LightGBM	learning_rate	[0.01, 0.1]	0.05	0.94	0.95	35
	num_leaves	[5, 50]	46			
	num_iterations	[20, 500]	195			
CatBoost	iterations	[1000, 5000]	4811	0.93	0.94	35
	learning_rate	[0.01, 0.1]	0.05			

Note. * The range of values of the parameter is defined on a logarithmic scale, i.e., the parameter values are uniformly distributed in logarithmic space. The random seed for hyperband parameter tuning is 111, and the eta is 2.
